# Physiological Striae Atrophicae of Adolescence with Involvement of the Upper Back

**DOI:** 10.1155/2013/386094

**Published:** 2013-07-15

**Authors:** Alexander K. C. Leung, Benjamin Barankin

**Affiliations:** ^1^Department of Pediatrics, the University of Calgary, the Alberta Children's Hospital, No. 200, 233–16th Avenue NW, Calgary, AB, Canada T2M 0H5; ^2^Toronto Dermatology Centre, Toronto, ON, Canada M3H 5Y8

## Abstract

We report a 13-year-old boy with multiple purplish, atrophic, horizontal linear striae in the thoracic area. He reported a growth spurt in the preceding 12 months. His past health was unremarkable, and he took no medications. To our knowledge, physiological striae atrophicae of adolescence where idiopathic striae were restricted to the upper back have rarely been reported. Physiological striae atrophicae of adolescence may, on occasions, be mistaken for child abuse. It is important that child care professionals recognize this condition so that false accusations of child abuse will not be made.

## 1. Introduction 

Physiological striae atrophicae of adolescence occurs mainly in healthy, nonobese individuals at around puberty in association with the adolescent growth spurt. The development of striae coincides with the markers of adolescence such as testicular enlargement, breast development, pubic hair growth, and menarche. By definition, there is no identifiable underlying cause such as an endocrine or connective tissue disorder. The condition is more common in boys, presumably because boys grow faster than girls at around puberty. The onset of striae is usually between 14 and 20 years of age in males and 10 and 16 years of age in females [1]. We describe a 13-year-old boy with physiological striae atrophicae of adolescence presenting with multiple transverse striae on the upper back. To our knowledge, physiological striae of adolescence where idiopathic striae were restricted to the upper back have rarely been reported.

## 2. Case Report 

A 13-year-old boy presented with multiple transverse striae on the upper back. The striae were first noted six months ago. There was no history of trauma, excessive physical exertion, or weight lifting. He was otherwise healthy and was not taking medications. The child had gained 10.9 kg and had grown 15.8 cm over the preceding 12 months. No family members had similar skin lesions.

**Figure 1:**
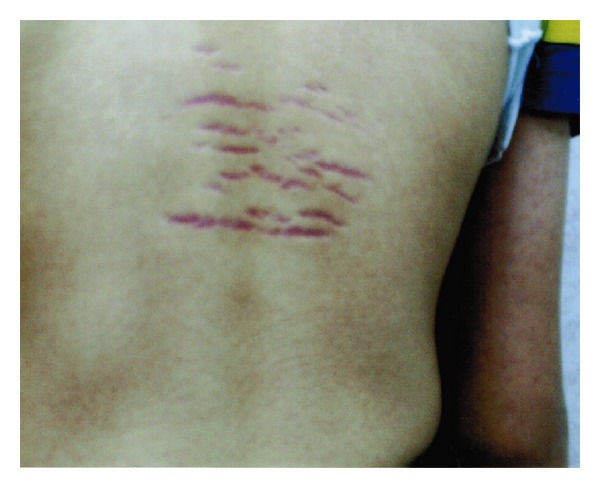
Multiple purplish, atrophic, horizontal linear striae on the upper back.

On examination, his weight was 55 kg (75th percentile) and height 160 cm (50th percentile). His heart rate was 68 beats per minute and blood pressure 110/70 mm Hg. Multiple purplish, atrophic, horizontal linear striae were noted at the back in the thoracic area (Figure 1). His pubic hair was in the Tanner stage 3 of development. He had testicular enlargement compatible with his chronological age. The penile size was normal for his age. He had some acne on his forehead. He also had axillary hair. The rest of the physical examination was unremarkable.

## 3. Discussion

Physiological striae atrophicae of adolescence typically presents as red or purple, horizontal, linear streaks (striae rubra) in the lumbar area [2]. Over time, the color fades and the lesions become atrophic and silvery (striae alba). They are usually several cm long and 1 to 10 mm wide, with the long axis perpendicular to the direction of skin tension [3]. Transverse striae of the back in adolescence were first reported by Parkes Weber in 1935 and then by Rosenthal in 1937 [4, 5]. Parkes Weber used the term “idiopathic” striae atrophicae of puberty to describe this condition [4]. There was a resurgence of interest in this condition in the 1960s. In 1964, Shelley and Cohen described a 16-year-old boy with a linear atrophic stria running transversely over a lower thoracic vertebra [6]. The child had been weight lifting and performing trampoline exercises during the preceding years; the authors believed that the stria represented a dermal tear due to the stress of repetitive weight lifting. In the following year, Savage reported a 16-year-old boy who was a weight lifter, with multiple similar striae on his back [7]. Nowadays, it is believed that mechanical shearing and stretching of the skin do not alone cause the striae. Hormonal (excessive cortisol level) and genetic factors are also operative. In 1968, Linn described a 18-year-old male with idiopathic transverse striae atrophicae of the lower back [8]. More recently, Feldman and Smith reported a 15-year-old boy with idiopathic striae atrophicae of puberty who presented with 12 purple linear markings in the lumbar area [9]. In a study of 1,037 college-age reserve officer training corps cadets in the United States, 56 (5.4%) were found to have atrophic striae running transversely across the back in the lumbosacral area [10]. In a study of 157 Korean students (109 boys and 48 girls) aged 15 to 17 years, striae were present in 94 (86.2%) boys and 37 (77.1%) girls [11]. Eighty-four (89.4%) boys and 31 (86.1%) girls had striae in the buttocks. In boys, the next common body region affected was the lower back (*n* = 26, 27.7%), followed by the knee (*n* = 23, 24.5%), calf (*n* = 21, 22.3%), upper arm (*n* = 15, 16%), thigh (*n* = 15, 16%), abdomen (*n* = 3, 3.2%), upper back (*n* = 2, 2.1%), and chest (*n* = 1, 1.1%). In girls, the thigh (*n* = 17, 45.9%) was the second most common body region affected, followed by the calf (*n* = 11, 29.7%), lower back (*n* = 7, 18.9%), knee (*n* = 3, 8.1%), abdomen (*n* = 2, 5.4%), and upper arm (*n* = 1, 2.7%). No stria was noted in upper back or chest. In contrast to other studies, children in this study had striae in multiple sites. This might be due to the differences in racial background and lifestyle of the study subjects. Physiological striae atrophicae of adolescence occurring in the thoracic area are rare, not to mention that these striae are present exclusively in the thoracic area. Our patient is unique in that the striae were restricted to the thoracic area.

In contrast to physiological striae atrophicae of adolescence, lesions of striae distensae or “stretch marks” occur mainly in areas that are subject to distension, such as the lower abdomen, lateral thighs, buttocks, and, in the females, the breasts. The condition occurs more frequently in females than males with a female-to-male ratio of 2.5 : 1 [2]. Striae distensae occur in association with a number of conditions such as obesity, pregnancy (striae gravidarum), prolonged use of systemic or topical corticosteroids, excessive use of marijuana, Cushing syndrome, and Marfan syndrome [12]. Striae distensae may also occur after breast augmentation or intense slimming diets [13].

Physiological striae of adolescence have to be differentiated from linear focal elastosis. The latter is characterized by asymptomatic yellow linear palpable bands, usually horizontally across the lower back.

Physiological striae of adolescence have been mistaken for nonaccidental injury. Heller reported two 13-year-old boys who were referred to the social services department because they had physiological striae manifested as horizontal linear marks in the lumbar and lumbosacral area, respectively [14]. Cohen et al. reported 4 adolescents with physiological striae; three of them with striae in the lumbar area were suspected by their school nurse to have been physically abused and the fourth child with striae in the lumbosacral area was suspected by his general practitioner as physical abuse [15]. Burk et al. reported a 15-year-old boy with violaceous, atrophic, horizontal striae on his lumbosacral region; the pediatrician of this child referred him to social services for investigation of child abuse [3]. Robinson et al. reported a 15-year-old boy with numerous striae on the back [16]. The child alleged that the striae might have been inflicted on him by his grandmother through her occult powers although he had not seen his grandmother for several years. A dermatologist was consulted and a diagnosis of striae distensae was made. Recently, Masand described a 16-year-old boy whose physiological striae on his back were suspected by the emergency department staff as a result of nonaccidental injury [17]. The child was later reviewed by a pediatrician who diagnosed these as physiological striae of adolescence. Thus, it is important for child care professionals be familiar with the benign nature of the condition in order to prevent false accusations of child abuse. This is especially so if the striae occur in the upper back—an unusual site for physiological striae of adolescence.
